# Circulating miRNA expression in long-standing type 1 diabetes mellitus

**DOI:** 10.1038/s41598-023-35836-8

**Published:** 2023-05-27

**Authors:** Paula Morales-Sánchez, Carmen Lambert, Jessica Ares-Blanco, Lorena Suárez-Gutiérrez, Elsa Villa-Fernández, Ana Victoria Garcia, Miguel García-Villarino, Juan Ramón Tejedor, Mario F. Fraga, Edelmiro Menéndez Torre, Pedro Pujante, Elías Delgado

**Affiliations:** 1 Endocrinology, Nutrition, Diabetes and Obesity Group (ENDO), Health Research Institute of the Principality of Asturias (ISPA), Av. Hospital Universitario s/n, 33011 Oviedo, Asturias Spain; 2 Centre for Biomedical Network Research on Rare Diseases (CIBERER), Instituto de Salud Carlos III, Madrid, Spain; 3 University of Barcelona, Barcelona, Spain; 4 Endocrinology and Nutrition Department, Asturias Central University Hospital, Oviedo, Asturias Spain; 5 Medicine Department, University of Oviedo, Oviedo, Asturias Spain; 6 Nanomaterials and Nanotechnology Research Center (CINN-CSIC), Health Research Institute of Asturias (ISPA), Oviedo, Asturias Spain; 7 Institute of Oncology of Asturias (IUOPA), Oviedo, Asturias Spain; 8 Department of Organisms and Systems Biology (B.O.S), University of Oviedo, Oviedo, Asturias Spain

**Keywords:** Endocrine system and metabolic diseases, Non-coding RNAs, Biomarkers

## Abstract

Type 1 diabetes is a chronic autoimmune disease which results in inefficient regulation of glucose homeostasis and can lead to different vascular comorbidities through life. In this study we aimed to analyse the circulating miRNA expression profile of patients with type 1 diabetes, and with no other associated pathology. For this, fasting plasma was obtained from 85 subjects. Next generation sequencing analysis was firstly performed to identify miRNAs that were differentially expressed between groups (20 patients vs. 10 controls). hsa-miR-1-3p, hsa-miR-200b-3p, hsa-miR-9-5p, and hsa-miR-1200 expression was also measured by Taqman RT-PCR to validate the observed changes (34 patients vs. 21 controls). Finally, through a bioinformatic approach, the main pathways affected by the target genes of these miRNAs were studied. Among the studied miRNAs, hsa-miR-1-3p expression was found significantly increased in patients with type 1 diabetes compared to controls, and positively correlated with glycated haemoglobin levels. Additionally, by using a bioinformatic approach, we could observe that changes in hsa-miR-1-3p directly affect genes involved in vascular development and cardiovascular pathologies. Our results suggest that, circulating hsa-miR-1-3p in plasma, together with glycaemic control, could be used as prognostic biomarkers in type 1 diabetes, helping to prevent the development of vascular complications in these patients.

## Introduction

Type 1 diabetes mellitus is a chronic autoimmune disease characterized by pancreatic beta cell deficiency, resulting in inefficient regulation of glucose homeostasis and lifelong insulin treatment need^1^. The most widely accepted theory attributes the development of type 1 diabetes to a combination of environmental, immunological, metabolic, and genetic stressors^2^. The influence of these stressors sustained over time can influence gene expression and post-transcriptional mechanisms, leading to overt autoimmunity, the development of type 1 diabetes, and ultimately, distinct comorbidities, highlighting vascular pathologies^2^.

Nowadays, the principal diabetes management guidelines use glycosylated haemoglobin (HbA1c), glucose, C-peptide and autoantibodies measurements for the diagnosis of type 1 diabetes^3^, however, it is still necessary to search for new parameters that broadens the understanding of the progression of the disease.

Regarding this, microRNAs (miRNAs) have appeared to be used as biomarkers, not only for the detection of different pathologies, but also to predict the prognosis of an already diagnosed disease. miRNAs are small non-coding RNA molecules (~ 22 nt) that can modulate gene expression post-transcriptionally, consequently controlling several biological processes^4^. Furthermore, they can be transported as stable molecules in body fluids, participating as mediators of cell-to-cell communication. In fact, miRNAs can reflect tissue damage, and their dysregulation has been linked to the development of different diseases^2,4^.

Although miRNA alterations have been studied in relation to diabetes^5–9^, there are many factors that may be confounding the results, leading to mixed and conflicting results: among these factors we can highlight the type of diabetes, disease duration, associated autoimmune diseases, body composition and metabolic control. The aim of this study is to analyze the expression profile of circulating miRNAs in the plasma of adult patients with well-established type 1 diabetes and with no associated comorbidities, compared to healthy subjects, to identify miRNAs that are specific from diabetes pathology, and to assess pathways and biological processes related to the state of this disease.

## Materials and methods

### Participants

This is an observational case–control study, carried out in adult patients who attended the Endocrinology and Nutrition Service of the Central University Hospital of Asturias, between June 2019 and December 2021. Written informed consent was obtained from all participants and the study was conducted in accordance with the principles of the Declaration of Helsinki for human research. The protocol was approved by the Ethical Committee at the Central University Hospital of Asturias (Project no. 32/19, March 2019, Oviedo, Asturias, Spain).

All patients with type 1 diabetes included in the study had been formally diagnosed according to ADA criteria^10^ and had at least 2 years of disease evolution. All participants passed a complete medical anamnesis, and were subjected to routine anthropometry, body composition and biochemical analysis.

The inclusion criteria for the patients included in the study were: no history of cancer, no other autoimmune diseases, no infections and/or catarrhal processes in the last 15 days before blood collection, no obesity [body mass index (BMI) ≥ 30 kg/m^2^], and no micro or macrovascular complications. In addition, to be included in the discovery cohort they had to fulfil the following criteria: at least one of the HLA positive (DR3, DR4, DQ2, DQ8), not taking statins or other medications instead of insulin, and not being a smoker. Regarding the control group, the inclusion criteria for control group were no history of cancer, no autoimmune diseases, no infections and/or catarrhal processes in the last 15 days before blood collection, no obesity and no vascular complications and glycated haemoglobin (HbA1c) ≤ 5.5%.

Finally, a total of 85 participants were enrolled and divided into two cohorts: discovery cohort (n = 30: 10 controls and 20 patients) and validation cohort (n = 55: 21 controls and 34 patients). To divide the two cohorts, HLA status was considered. Since the presence of certain HLAs are a known risk factor for type 1 diabetes and autoimmune diseases, we aimed to select controls in the discovery cohort who did not have any of the specific HLAs associated with the disease (DR3, DR4, DQ2, DQ8) to minimize the risk of including individuals with a genetic predisposition for developing type 1 diabetes or autoimmune diseases. However, these HLAs are prevalent in the general population, individuals in the validation cohort who were healthy and were positive for any of them were included to expand the control group. Clinical characteristics of the participants enrolled in the present study are reported in Table 1.

**Table 1:** Demographical and biochemical characteristics of study cohorts.

	Discovery cohort		Validation cohort	
	Control	Type 1 diabetes	Control	Type 1 diabetes
N	10	20	21	34
Gender (% male)	50	65	29	62
Age (years)	34.6 ± 3.0	33.2 ± 2.3	28.9 ± 2.1	34.4 ± 2.2
Age at onset (years)	–	16.6 ± 3.1	–	19.7 ± 2.0
Disease duration (years)	–	16.0 ± 2.3	–	14.7 ± 1.4
Anthropometric				
BMI (kg/m^2^)	24.0 ± 0.8	24.9 ± 0.7	22.2 ± 0.4	24.1 ± 0.6**
Fat (%)	25.5 ± 2.4	23.3 ± 2.2	25.6 ± 1.8	23.6 ± 1.4
Waist	80.1 ± 2.9	84.6 ± 2.4	75.0 ± 2.0	84.5 ± 2.4*
Biochemical				
Glucose (mg/dL)	82.6 ± 1.9	187.0 ± 15.8***	80.6 ± 1.6	200.0 ± 14.4***
HbA1c1 (%)	5.0 ± 0.1	7.0 ± 0.2***	4.9 ± 0.1	7.7 ± 0.2***
C Peptide (ng/mL)	–	0.22 ± 0.05	–	0.14 ± 0.07
Total cholesterol (mg/dL)	182.0 ± 13.6	178.0 ± 6.8	169.8 ± 5.9	175.4 ± 5.3
HDL (mg/dL)	62.0 ± 5.3	59.7 ± 1.8	65.1 ± 2.9	63.6 ± 2.7
Triglycerides (mg/dL)	69.3 ± 6.5	80.1 ± 6.6	66.9 ± 4.8	82.8 ± 7.8
LDL (mg/dL)	106.0 ± 12.1	102.0 ± 6.0	91.3 ± 5.0	96.2 ± 4.5
TSH	2.4 ± 0.2	2.5 ± 0.3	2.3 ± 0.2	2.3 ± 0.2
HLA				
HLADR3 (%)	0	100	28.6	58.8
HLADR4 (%)	0	85	23.8	50.0
HLADQ2 (%)	0	100	28.6	58.8
HLADR8 (%)	0	80	23.8	47.1

Data are expressed as mean ± SEM. Values for categorical variables are showed in %

*TSH* thyroid stimulating hormone, *BMI* body mass index, *HDL* hight-density lipoprotein, *LDL* low-density lipoprotein.

Significance: Comparisons with controls: *p* values < 0.05 (*), < 0.01 (**), < 0.001 (***).

### Anthropometric measures

All patients were subjected to routine anthropometric examinations including body weight, height, and waist circumference measurements. Body composition studies were carried out by using Bioelectrical Impedance Analysis technology (Tanita, T5896 Tokyo, Japan), which is a validated method to determine total and segmental composition.

### Blood collection and sample preparation

Overnight fating peripheral blood samples from all subjects were collected in EDTA-containing Vacutainer tubes (BD Biosciences). Blood samples were immediately centrifuged at 2000× rpm for 15 min at 4 °C. The top layer containing the plasma was divided into aliquots and stored at − 80 °C until further analysis.

### Illumina small-RNA sequencing in the screening

Small-RNA isolation, library preparation, quality control and next generation sequencing procedures were accomplished by Novogene Co., Ltd (Cambridge, United Kingdom). Total RNA was isolated from 200 µL of plasma using the miRNeasy Serum/Plasma kit (Qiagen, Germany) in accordance with the manufacturer’s recommendations. 2 µg of total RNA per sample were used as input material for small-RNA cDNA libraries generation using NEBNext Multiplex Small RNA Library Prep Set for Illumina® (New England Biolabs, United States), following the manufacturer’s instructions. After, library quality was assessed on the Agilent Bioanalyzer 2100 system (Agilent Technologies, Santa Clara, CA, USA). Finally, 50 bp single-end reads were generated on an Illumina Novaseq 6000 platform (Illumina, CA, USA).

### miRNAs bioinformatic data analysis

Raw sequencing data quality was evaluated using FastQC software (0.11.9)^11^ Cutadapt (3.4) was used to trim full and truncated adapter sequences and set a minimal sequence length of 17 nucleotides^12^. Alignment on hg38 human genome reference was performed using Bowtie1 (1.0.0) and annotation and read counting of predicted miRNAs from miRbase v22.1 applying mirDeep2 (2.0.1.2) package^13,14^. The small RNA that is obtained through miRDeep2 are mainly the mature sequences, which are eventually produced by more than one precursor throughout the genome. Therefore, all mature miRNAs with the same names were taken from different precursors and averaged for each (rounded up).

In order to identify miRNAs that were differentially expressed, read counts were analysed using edgeR (v.3.36.0) package from Bioconductor in R environment (v.4.1.3)^15^. miRNA data normalization was performed using the with the trimmed mean of M values method (TMM) method and calculated the effective library size. miRNAs were considered detectable if they had expression levels of > 1 counts per million (CPM) in more than half of the samples in the discovery cohort (E-MTAB-12641). Subsequently, differential expression analysis used the quasi-likelihood negative binomial generalized log-linear model (GLM) functions provided by the edgeR package. Statistical significance for differentially expressed miRNA was defined as *p* values < 0.05, and absolute log2 fold changes ≥ than 1. In addition, only differential expressed miRNAs with an average log_2_ counts per million (logCPM) greater than 5 were considered for subsequent validation.

### miRNAs housekeeping selection

miRNA expression stability data from the entire cohort for housekeeping gene selection were analysed using RefFinder^13^, which can be used to confirm and integrate the output obtained from the comparative algorithms Delta-CT, geNorm, NormFinder, and BestKeeper. RefFinder produces a final overall ranking of the reference genes under evaluation based on the geometric mean calculated from the weight of each gene produced by each of the individual algorithms. Finally, regarding manufacture’s instruction and literature, hsa-miR-191-5p was selected as housekeeping.

### RNA isolation and quantification in the validation cohort

Total RNA was isolated from 200 µL of frozen plasma samples in silica membrane columns using the miRNeasy Serum/Plasma Advanced Kit (Qiagen, Hildem Germany) according to the manufacturer’s instructions. The mixture was supplemented with 1.5 µg of bacteriophage MS2 carrier RNA (Roche, Merck, Darmstadt, Germany) to improve isolation yield. RNA was finally eluted into 20 µL of nuclease-free water and stored at − 80 °C until further use.

Isolated total RNA was reverse transcribed into cDNA using the TaqMan advanced miRNA cDNA synthesis kit (Life Technologies, California, USA). Gene expression analysis was carried out by RT-PCR using TaqMan® Gene Expression assays (Applied Biosystems; Suppl. Table S1) and the Applied Biosystems Prism 7900HT Sequence Detection System (Applied Biosystems) according to manufacturer’s instructions. Gene expression data are expressed as target gene mRNA expression relative to the corresponding housekeeping mean gene expression (ΔC_T_ = C_T_ miRNA—geometric mean C_T_ value of the housekeeping miRNA). The relative expression of each miRNA was reported as 2^−ΔCT^^16^.

### Putative gene targets, pathway enrichment analysis and related diseases

Target network, functional and pathway enrichment analysis were all performed using miRNet web-based tool^17^. miRbase IDs for the differentially expressed miRNAs were uploaded for each comparison. Subsequently, miRTarBase v8.0 database was selected to annotate experimentally supported target miRNA genes^18^. Since circulating plasma miRNA was being analysed, no tissue background was selected.

For functional evaluation, enrichment analysis was conducted using the Kyoto Encyclopedia of Genes and Genomes (KEGG)^19^ implemented in miRNet software and represented using RStudio tools. In addition, a hypergeometric test was performed to identify the biological significance of the genes among the identified targets. Only statistically significant annotation categories were retained (*p* value < 0.05).

Associated pathologies related to the selected miRNAs was carried out by looking for indexed publications. Therefore, the Human MicroRNA Disease Database (HMDD v3.2; updated march-27, 2019) was used^20^ which is a database that provides curated experiment-supported evidence for human miRNA and disease associations.

### Statistics

Statistical differences between controls and groups of patients were analysed by the Wilcox test (for two groups) or Kruskal–Wallis test (for three or more groups) with Dunn test for post hoc comparisons as appropriate. Pearson r-rank correlation coefficient matrix for miRNA expression and the different clinical parameters was computed using *rcorr* function in *Hmisc* package (v.4.7-0). All statistical analyses were performed in R environment (v.4.1.3).

### Institutional review board statement

This study was approved by the Ethical Committee at the Central University Hospital of Asturias (Project no. 32/19, March 2019, Oviedo, Asturias, Spain) and conducted in accordance with the principles of the Declaration of Helsinki for human research.

## Results

### Plasma circulating miRNA expression profile in type 1 diabetes

**Figure 1:**
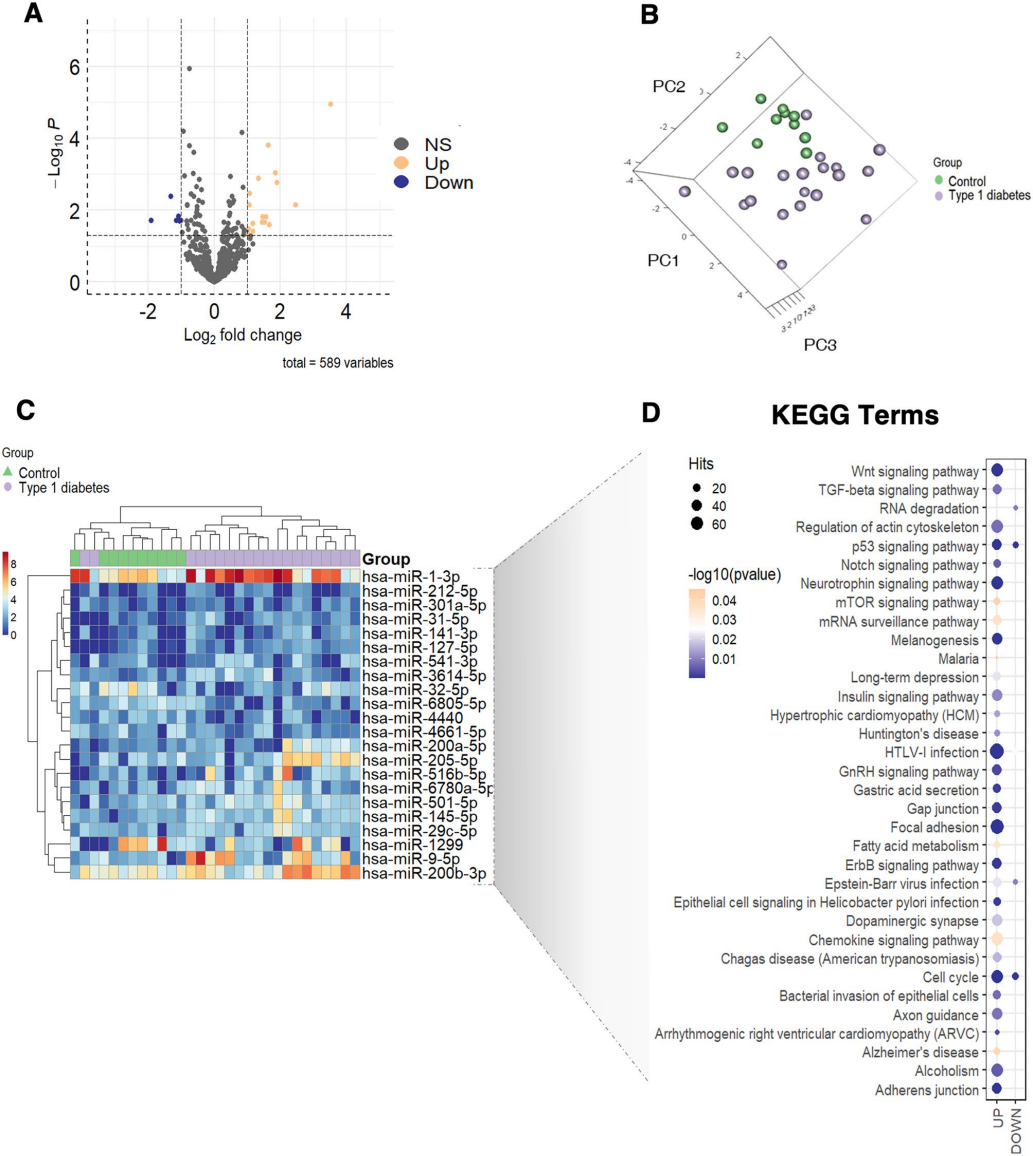
Plasma miRNA profile by next-generation sequencing (NGS) in the discovery cohort. (**A**) Volcano plot showing the 589 variables detected by NGS. Orange dots (up) refer to overexpressed miRNAs and dark blue dots (down) to downregulated miRNAs according to the differential expression criteria described (log2FC greater than 1 and less than − 1, with a *p* value less than 0.05). (**B**) 3D-principle component analysis (3D-PCA) of circulating miRNA expression in the discovery cohort. 3D-PCA decomposition of the 22 differential expressed miRNAs could distinguish most diabetes (purple) cases from the control (green) group in the NGS discovery cohort. (**C**) Heatmap representation of the expression levels of the 22 differential miRNAs expressed in log_2_CPM. (**D**) KEGG dotplot showing pathway terms with *p* value less than 0.05 for genes that are predicted in miRNet as controlled by upregulated (UP) and downregulated (DOWN) miRNAs.

This study was divided into two different phases. First, we performed a discovery phase, to identify the plasma miRNA transcriptome differences between the study groups, by next-generation small-RNA sequencing approach. Of the 2656 known miRNAs for the species annotated in miRBase (release 22)^14^, after quality control and filtering of duplicates, 1679 were found with one read in at least one patient of the cohort. Following threshold application for non-detectable miRNAs and data normalisation, 589 were found in our discovery cohort. Subsequently, analyses were carried out to investigate differentially expressed miRNAs in the data sets for all the comparisons. The criterium for differential expression was established as log_2_ fold change (logFC) ≥ 1 or ≤ − 1 and *p* value < 0.05. Patients with type 1 diabetes exhibited a total of 84 miRNAs with significant *p* values, compared to controls. Of them, 22 met the established differential expression criteria, being 5 miRNAs down-regulated and 17 up-regulated (Table 2; Fig. 1A). 3-dimensional principal component analysis (3D-PCA) of the logCPM values of differentially expressed miRNAs were represented (Fig. 1B). Hierarchical clustering was also performed on these miRNAs and displayed as a heat map (Fig. 1C). Additionally, based on the Kyoto Encyclopedia of Genes and Genomes (KEGG), pathway analysis for the miRNA target genes was finally performed. For this, all predicted target genes, but without including cancer-related terms were used. 32 pathways were found to be affected by genes controlled by upregulated miRNAs, while only 5 pathways were controlled by downregulated miRNAs, being 4 of these pathways shared with the upregulated group (Fig. 1D; Suppl. Fig. S1).

**Table 2:** Differentially expressed miRNAs detected by NGS in plasma of type 1 diabetes patients vs controls in the discovery cohort.

miRNA	logFC	logCPM	*p* value	FDR
hsa-miR-9-5p	3.51005764	5.09554677	1.11E−05	0.00327952
hsa-miR-516b-5p	2.44926464	3.40713745	0.00713453	0.13715332
hsa-miR-200a-5p	1.89070648	2.66074451	0.00172292	0.07248585
hsa-miR-1-3p	1.84328428	7.42754601	0.00091122	0.06109701
hsa-miR-212-5p	1.66621456	1.22220223	0.02498261	0.24524597
hsa-miR-145-5p	1.62687465	3.04240094	0.00015271	0.01581594
hsa-miR-541-3p	1.56140602	2.11900999	0.01523402	0.21079678
hsa-miR-141-3p	1.52010471	1.58771785	0.02185397	0.24524597
hsa-miR-31-5p	1.46156545	1.593171	0.01514239	0.21079678
hsa-miR-205-5p	1.44554199	3.88685627	0.02167744	0.24524597
hsa-miR-200b-3p	1.32981486	5.35549997	0.00129151	0.06264239
hsa-miR-127-5p	1.1710895	1.45032739	0.03936907	0.29083546
hsa-miR-3614-5p	1.17082868	2.28774798	0.02365133	0.24524597
hsa-miR-29c-5p	1.06292869	2.81232099	0.0033877	0.10757119
hsa-miR-501-5p	1.05522169	3.12288162	0.00701167	0.13715332
hsa-miR-6780a-5p	1.03618222	2.80218622	0.03318434	0.29083546
hsa-miR-301a-5p	1.01865161	1.31268582	0.04672534	0.31633593
hsa-miR-4661-5p	− 1.05250628	2.218599	0.01967106	0.2413803
hsa-miR-32-5p	− 1.08223575	3.11166773	0.01480409	0.21079678
hsa-miR-4440	− 1.15245103	1.94161952	0.01931516	0.2413803
hsa-miR-6805-5p	− 1.3165362	2.39204186	0.00418125	0.1119435
hsa-miR-1299	− 1.90245644	4.95517825	0.01929258	0.2413803

**Figure 2:**
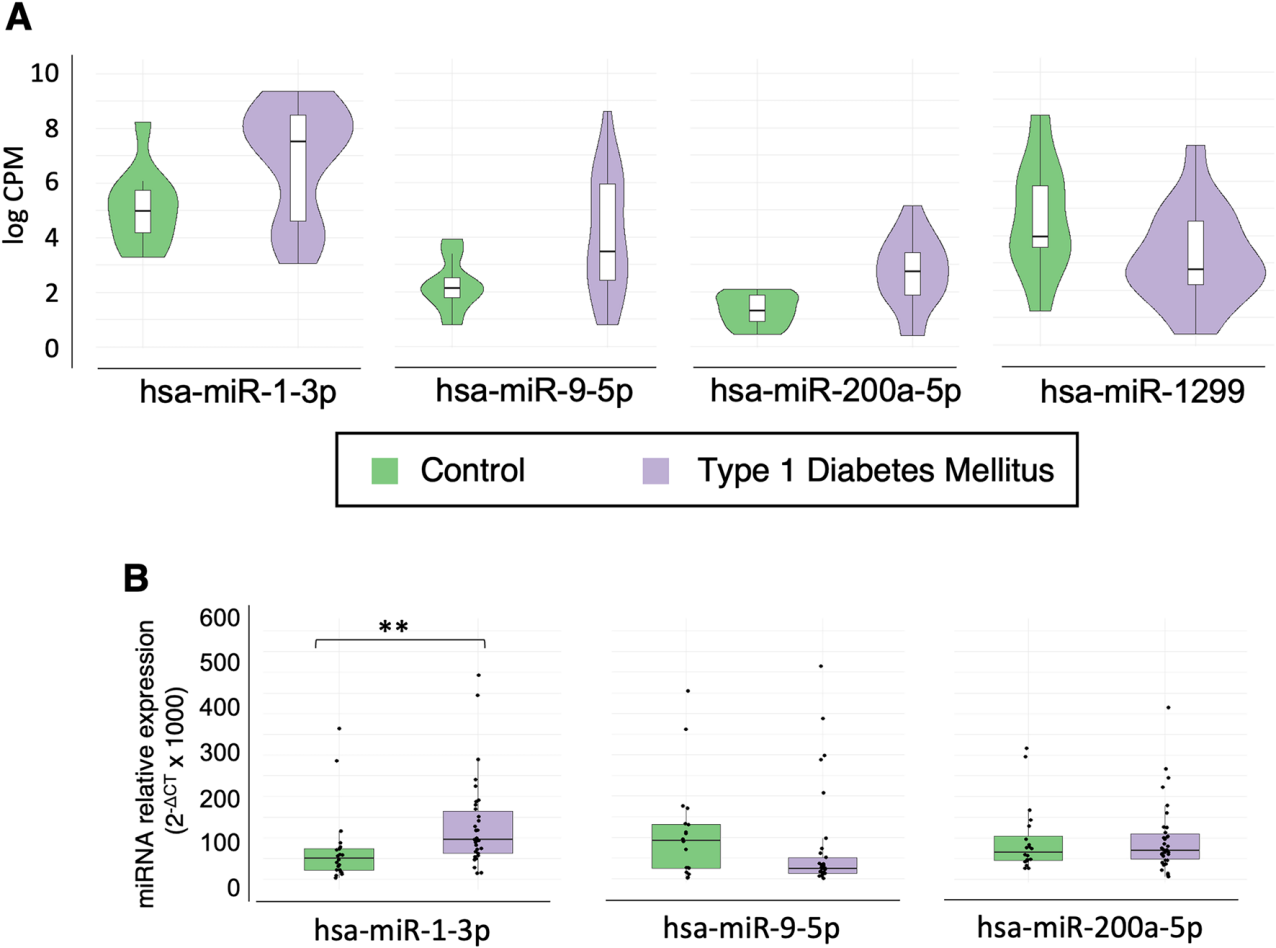
Box plots of differentially expressed circulating miRNAs in the discovery cohort using NGS approach. (**A**) NGS log CPM expression of selected miRNAs. (**B**) RT-PCR expression of NGS selected miRNAs. Significance: *p* values < 0.05 (*), < 0.01 (**), < 0.001 (***).

Among the 22 differentially expressed miRNAs, those miRNAs with logCPM values greater than 5 were selected for its validation in a new cohort of 55 volunteers (21 controls, and 34 type 1 diabetic patients). Only four miRNAs complied with that criterium: hsa-miR-1-3p, hsa-miR-200b-3p and hsa-miR-9-5p, were overexpressed in the type 1 diabetes group and hsa-miR-1299 downregulated in the type 1 diabetes group from the discovery cohort (Fig. 2A). For this validation, miRNA expression was measured by real time PCR with Taqman probes.

Starting from the logCPM of the NGS data, the miRNA normalizers were chosen using the RefFinder tool. Taking into account the results obtained with the program (Suppl. Fig. S2A) and the available bibliography^21–23^, hsa-let-7d-5p, hsa-miR-191-5p and hsa-miR-24-3p miRNAs were selected. Subsequently, miRNA expression levels were also measured by RT-PCR, and verified with the same program if they could be used as normalizers. The results (Suppl. Fig. S2B–D; Suppl. Table S2) showed that in the ranking, hsa-miR-191-5p was the most stable miRNA, and thus, it was selected for RT-PCR normalization.

Among the four miRNAs selected for its analysis in the validation cohort, hsa-miR-1299 was excluded since more than the half of the samples could not be detected by RT-PCR. Regarding the other three, only hsa-miR-1-3p showed the significant upregulation expected in the type 1 diabetes group. In contrast, no change in expression was observed in either hsa-miR-200b-3p or hsa-miR-9-5p (Fig. 2B).

### Gene target prediction and pathway enrichment analysis

**Figure 3:**
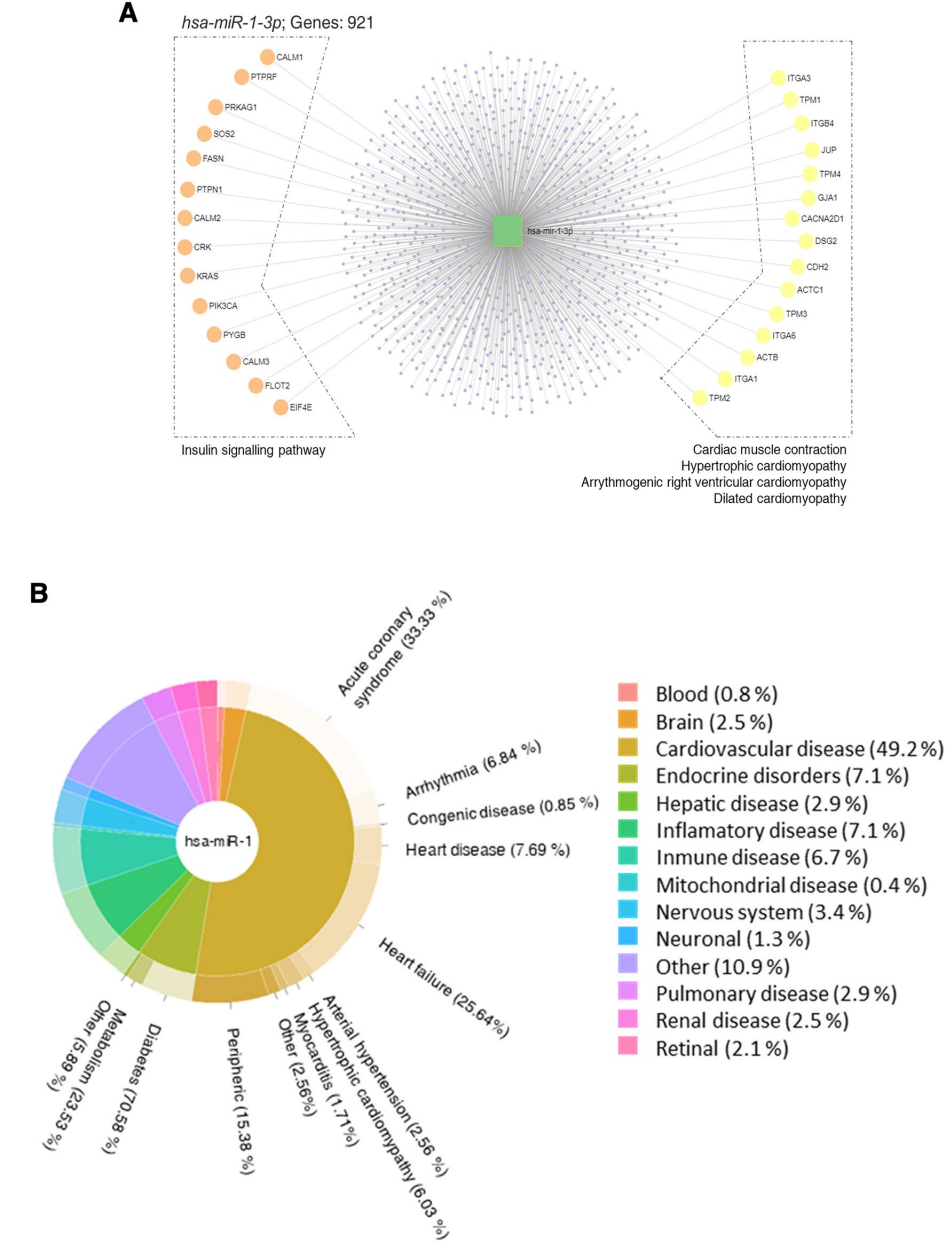
(**A**) hsa-miR-1-3p predicted target genes and the KEGG pathways in which they are involved. miRNAs’ network of targets predicted by miRNet**.** (**B**) PieDonut plots referencing the number of disease-related terms in hsa-miR-1-3p. Curated experiment-supported evidence for human miRNA and disease associations in the Human microRNA Disease Database. The inner circumference represents the percentage of terms found for each miRNA with respect to the total. While the external one shows the percentages of each major term related to a disease within the miRNAs.

In addition to the KEGG pathways found to be potentially altered by differentially expressed miRNA gene targets by NGS approach (Fig. 1D), a new analysis was performed focusing exclusively on those routes putatively altered by hsa-miR-1-3p, which included cardiac muscle contraction and pathways related to cardiomyopathy (Fig. 3A). In addition, curated annotations for experimentally supported evidence for human miRNA and disease associations was retrieved from HMDD. By removing all terms related to oncologic research, that could be hiding important information about other pathologies, we could observe that almost 50% of the disease annotations on which hsa-miR-1-3p could be involved, were related to the cardiovascular system. Additionally, terms related to endocrine disorders, and inflammatory and immune diseases were also noted (Fig. 3B).

### Plasma circulating miR-1-3p expression is correlated with HbA1c1

**Figure 4:**
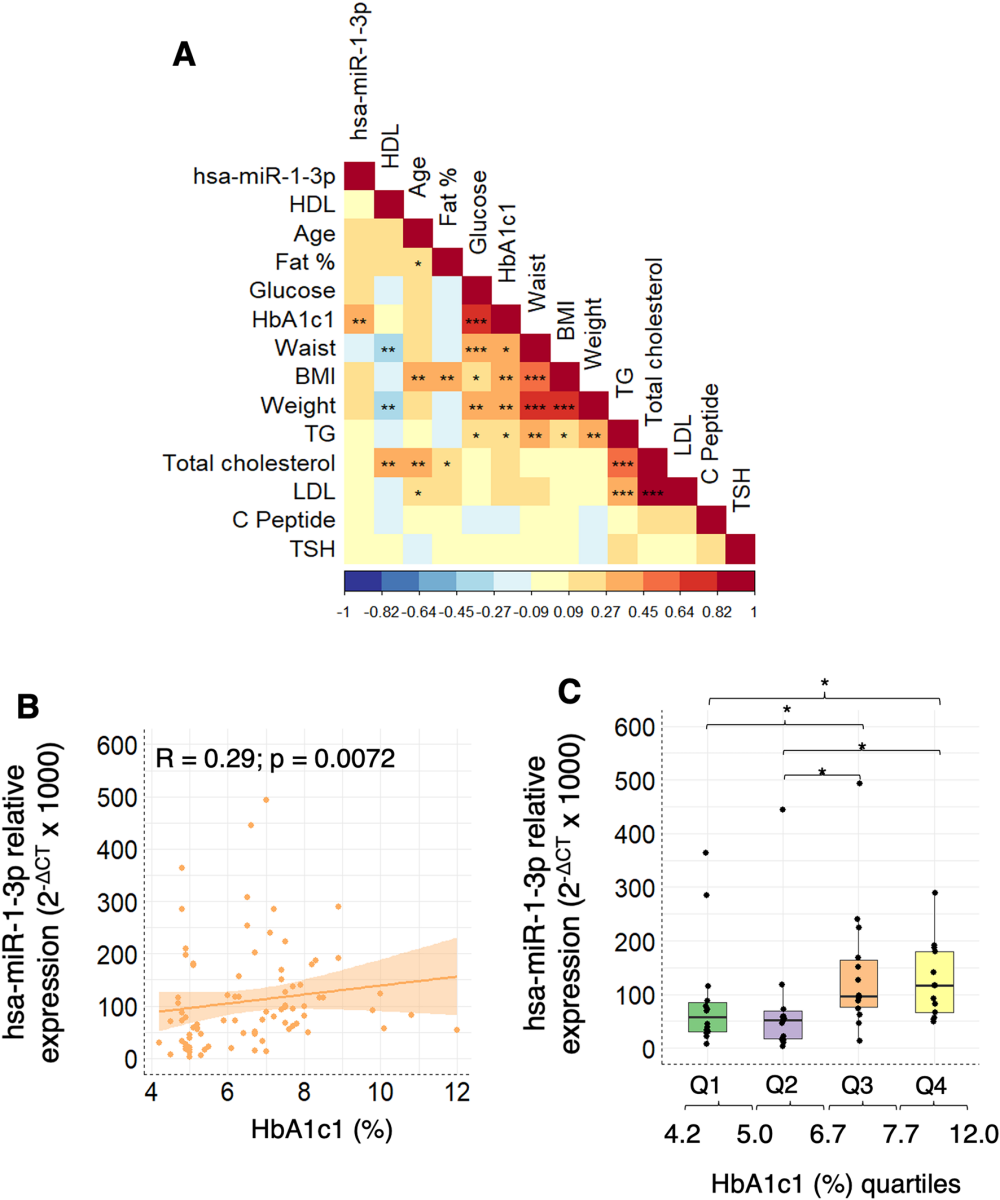
(**A**) Matrix correlation plot between hsa-miR-1-3p and clinical parameters for all samples (n = 55) included in the validation cohort. (**B**) Linear correlation plot between hsa-miR-1-3p and HbA1c percentage. (**C**) hsa-miR-1-3p expression among the different HbA1c1 quartiles. Significance expressed as: < 0.05 (*), < 0.01 (**) or < 0.001 (***).

To further assess the role of hsa-miR-1-3p in diabetic disease, correlations between miRNA expression data, biochemical blood anthropometric parameters were performed in the validation cohort. All data was collected at the same point for both patients and controls. A positive correlation between hsa-miR-1-3p and HbA1c1 values was observed (Fig. 4A,B).

Additionally, all volunteers in the validation cohort were grouped by HbA1c1 quartiles. Differences were only observed in the third and fourth quartiles as compared to the first quartile, but not with the second quartile in any of the contrasts (Fig. 4C). Given that in our cohort normal levels of glycosylated haemoglobin were established as those below 5.5%, we can assume that the patients with better glycaemic control showed a similar expression of hsa-miR-1-3p than the control group, while HbA1c1 higher than 6.7%, are associated with a higher expression of hsa-miR-1-3p.

## Discussion

The role of miRNAs in the development, diagnosis and prognosis is increasingly being studied in many fields, including in type 1 diabetes^24^. However, most of the studies based on the miRNA profile of patients with diabetes were performed on type 2 diabetes cohorts^9,25^. Regarding type 1 diabetes, the bulk of the research studying circulating miRNAs has been carried out in newly diagnosed paediatric patients^8,26–28^. Specifically, more than 200 miRNAs have been studied in different tissues, cells and blood samples, in the field of type 1 diabetes^7^. However, depending on the characteristics of the cohorts analysed, the dysregulated miRNAs may change and thus, expanding these studies to encompass diverse populations, including those with long-standing diabetes, could provide additional insights into the role of miRNAs in disease progression and guide the development of targeted therapeutic interventions.

It is also important to notice that, not only glucose levels, but also weight and body mass composition are important in the prognosis of type 1 diabetes mellitus^29,30^. However, type 1 diabetes has been described for years associated with normal weight, and there are not many studies that investigate the relationship of this pathology with the patient's body composition. For this reason, and to avoid possible interference between external and internal factors in the expression of circulating miRNAs, in this study we decided to analyse total body mass composition and exclude from the study those patients with any type of obesity or those who suffer from any complication associated with type 1 diabetes. In addition, C-Peptide was measured in all patients to confirm beta-cell dysfunction, so we can guarantee that the circulating miRNAs expression changes observed are due to diabetes disease, and that no other factor is involved.

In the present study, by using Next Generation Sequencing approach, we found a total of 22 miRNAs dysregulated in the long-standing type 1 diabetes group, compared with normoglycemic controls (5 downregulated and 17 upregulated). As expected, our study revealed the presence of certain miRNAs, including members of miR-200 family (miR-200a, miR-200b and miR-141)^31^, hsa-miR-9-5p^32,33^, and hsa-miR-212-5p^34^, among others, which have been previously reported to be implicated in beta cell function in diabetes mellitus, irrespective of the type. However, a noteworthy observation was the absence of other miRNAs such’s as miR-375, despite its widespread mention in literature^35,36^. At this point it is important to state that, the development of well-established type 1 diabetes, where patients are continuously treated with insulin due to the absence of beta cell reservoirs, may differ from the onset of the disease, and more importantly, from other types of diabetes. As a result, different miRNA expression profile could be associated with different disease stages.

Among the differentially expressed miRNAs, four were selected for their validation in an extensive cohort: hsa-miR-1-3p, hsa-miR-200b-3p, hsa-miR-9-5p, and hsa-miR-1299. For technical and biological validation, real time PCR was performed, and only hsa-miR-1-3p showed the expected significant upregulation in the type 1 diabetes patient group with this technique. miR-1-3p is a member of the *myomiR* family. It is expressed in both heart and skeletal muscle and is frequently seen in the development of diseases related to these tissues^37^. A recent study demonstrates that serum levels of cardiomyocyte-enriched miR-1 are predictors of myocardial steatosis in both in vitro and in patients with well-controlled and uncomplicated type 2 diabetes of short duration^38^. Another study showed that miR-1 and miR-133 together achieved good diagnostic performance for cardiovascular disease in type 2 diabetes, with miR-1 alone having even better diagnostic value^39^. Moreover, individuals with pre‑diabetes exhibited significantly higher expression levels of miR‑1 compared with the controls^40^.

Although there are certain aspects of type 2 diabetes that are similar to the autoimmune form of the disease, scarce information is available on how this miRNA might be involved in the development of the type 1 diabetes, in fact, it has mainly been described in cardiovascular comorbidities^41,42^. In this line, miR-1-3p has been found to be reduced in diabetic cardiomyocytes in a rat model of type 1 diabetes^43^ and also reduced in retinal, cardiac, and renal tissues from streptozotocin-induced diabetic mice^44^. Our results suggest that miR-1 may be altered at different stages of diabetes development, possibly playing distinct roles in the pathophysiology of the disease and its complications. To our knowledge, this is the first time that miR-1-3p has been reported to be upregulated in plasma of patients with long-standing type 1 diabetes, but without any cardiovascular complication. More interestingly, we have observed that miR-1-3p expression is positively correlated with HbA1c1 levels. HbA1c1 is considered as a diabetes control marker, being also related with the development of different complications in type 1 diabetes patients, including micro and macrovascular complications^45,46^. We hypothesize that, not only HbA1c1 levels, but also circulating miR-1-3p expression, could be taken into consideration in future analysis of vascular risk markers, in patients with type 1 diabetes mellitus. In fact, by excluding the impact of body weight on the expression of circulating miRNA, we can say that the higher expression of miR-1-3p could reflect a higher glycaemic load, which is the main factor involved in the development of vascular complications. Therefore, further research is necessary to elucidate the precise mechanisms underlying the role of miR-1 in type 1 diabetes development, including the factors that modulate its expression, and to explore its potential as a therapeutic target for diabetes-related complications. As limitation to our study, it is relevant to point out that is not uncommon to observe differences in miRNA profiles between the discovery and validation cohorts. Moreover, it should be noted that the deregulated miRNAs can vary depending on the characteristics of the analysed cohorts. These discrepancies between cohorts or samples can be attributed to various factors, such as demographic differences, variations in sample collection and processing, and measurement technique differences. PCR-based approaches and NGS, for example, have different sensitivities and specificities. To address these discrepancies, future studies could investigate larger sample sizes and employ standardized normalization techniques and data analysis methods for miRNA. This approach may reduce variability and improve reproducibility.

On the other hand, bioinformatic analysis was also performed to find the main target genes of all miRNAs significantly modified in the NGS analysis, as well as the annotated genes for miR-1-3p exclusively. We could then support the importance of these observed alterations, not only in glucose homeostasis, since the insulin signalling pathways has shown to be clearly affected, both in the NGS and the miR-1-3p analysis. But also in the vascular status of these patients, since important routes such as *Wnt* and *Notch signalling pathways* are both related with the processes of angiogenesis and atherosclerosis^47–49^. The Notch pathway was described to be involved in cell-fate determination during development, maintenance of adult tissue homeostasis, and also in the adipogenesis process in type 2 diabetes rats^50^, reinforcing the importance of body mass composition in diabetes pathology.

## Conclusion

Our results suggest that circulating miRNA, specifically hsa-miR-1-3p, could be implemented as biomarkers for the prediction and progression of type 1 diabetes, in the development of vascular complications. However, it would be necessary to deepen the analysis to determine the scope of this miRNA plasmatic alteration in patients with type 1 diabetes and its relationship with different clinical complications. In addition, it will be important to monitor the patients included in this study, which will be performed every 5 years, to be able to elucidate whether those subjects with higher hsa-miR-1-3p values eventually develop any vascular comorbidity associated with the diabetes pathology, as expected by our bioinformatic approach. Although more studies should be carried out, including a new group of patients with vascular pathology, such as patients with myocardial infarction, these findings could have an important clinical relevance and suggesting new diagnostic and therapeutic avenues for preventing future comorbidities in type 1 diabetes mellitus patients.

## Supplementary Information

Supplementary Information.

## Data Availability

The datasets generated during and/or analysed during the current study are available in ArrayExpress repository; Accession code: E-MTAB-12641 https://www.ebi.ac.uk/biostudies/arrayexpress/studies/E-MTAB-12641?key=1b2bcb8d-773a-4bfb-aaf5-8b70b58767f2.
