# Supplementary material for: Circulating miRNA expression in long-standing type 1 diabetes mellitus
Source: Sci Rep. 2023 May 27;13:8611. doi: 10.1038/s41598-023-35836-8 (PMC10224919; doi:10.1038/s41598-023-35836-8)
Supplement: Supplementary file 1 — Supplementary Information. [file 41598_2023_35836_MOESM1_ESM.docx]

Supplementary Material

**Circulating miRNA expression in long-standing type 1 diabetes mellitus**

Paula Morales-Sánchez^1,2,§^, Carmen Lambert^1,3,§^, Jessica Ares-Blanco^1,4,5^, Lorena Suárez-Gutiérrez^1,4^, Elsa Villa-Fernández^1^, Ana Victoria Garcia^1^, Juan Ramón Tejedor^2,6,7,8^, Mario F. Fraga^2,6,7,8^, Edelmiro Menéndez Torre^1,2,4,5^, Pedro Pujante^1,4, ‡^, and Elías Delgado^1,2,4,5,‡,^

^1^ Endocrinology, Nutrition, Diabetes and Obesity Group, Health Research Institute of the Principality of Asturias (ISPA), Oviedo, Asturias, Spain.

^2^ Centre for Biomedical Network Research on Rare Diseases (CIBERER), Instituto de Salud Carlos III, Madrid, Spain.

^3^ University of Barcelona, Barcelona, Spain.

^4^ Asturias Central University Hospital, Endocrinology and Nutrition Department, Oviedo, Asturias, Spain.

^5^ Medicine department, University of Oviedo, Oviedo, Asturias, Spain

^6^ Nanomaterials and Nanotechnology Research Center (CINN-CSIC), Health Research Institute of Asturias (ISPA), Oviedo, Asturias, Spain.

^7^ Institute of Oncology of Asturias (IUOPA), Asturias, Spain

^8^ Department of Organisms and Systems Biology (B.O.S), University of Oviedo, Oviedo, Asturias, Spain

§ Both authors contribute equally

‡ Both authors contribute equally

**Corresponding Authors:**

Carmen Lambert: lambert.goitia@gmail.com

Elias Delgado: eliasdelga@gmail.com

Pedro Pujante: [pedropujanteal@gmail.com](mailto:pedropujanteal@gmail.com)

| **Supplementary Table 1.** Taqman miRNA expression assays | | | |  |
| --- | --- | --- | --- | --- |
| **miRNA** | **TaqMan Reference** | **Sequence** | **Observation** | **Mean CT** |
| hsa-miR-191-5p | 477952_mir | 5´- CAACGGAAUCCCAAAAGCAGCUG-3´ | Housekeeping | 23.3 |
| hsa-miR-200b-3p | 477963_mir | 5´-UAAUACUGCCUGGUAAUGAUGA-3´ | Selected candidate | 26.6 |
| hsa-miR-1-3p | 477820_mir | 5 ́-UGGAAUGUAAAGAAGUAUGUAU-3 ́ | Selected candidate | 26.5 |
| hsa-miR-9-5p | 478214_mir | 5´-UCUUUGGUUAUCUAGCUGUAUGA-3´ | Selected candidate | 25.9 |
| hsa-miR-1299 | 478696_mir | 5´- UUCUGGAAUUCUGUGUGAGGGA -3´ | Selected candidate | - |

| **Supplementary Table 2.** miRNA housekeeping ranking order generated by RefFinder. | | | | | | | |
| --- | --- | --- | --- | --- | --- | --- | --- |
| **Ranking Order (Better--Good--Average)** | | | | | | | |
| **Method** | **1** | **2** | **3** | **4** | **5** | **6** | **7** |
| **Delta CT** | hsa-miR-191-5p | hsa-let-7d-5p | has-miR-24-3p | has-miR-200a-3p | hsa-miR-1-3p | has-miR-200b-3p | hsa-miR-9-5p |
| **BestKeeper** | hsa-miR-191-5p | has-miR-24-3p | hsa-let-7d-5p | has-miR-200b-3p | has-miR-200a-3p | hsa-miR-1-3p | hsa-miR-9-5p |
| **Normfinder** | hsa-miR-191-5p | hsa-let-7d-5p | has-miR-200a-3p | has-miR-24-3p | hsa-miR-1-3p | has-miR-200b-3p | hsa-miR-9-5p |
| **Genorm** | hsa-miR-191-5p \| \| has-miR-24-3p |  | hsa-let-7d-5p | has-miR-200b-3p | has-miR-200a-3p | hsa-miR-1-3p | hsa-miR-9-5p |
| **Recommended comprehensive ranking** | hsa-miR-191-5p | has-miR-24-3p | hsa-let-7d-5p | has-miR-200a-3p | has-miR-200b-3p | hsa-miR-1-3p | hsa-miR-9-5p |


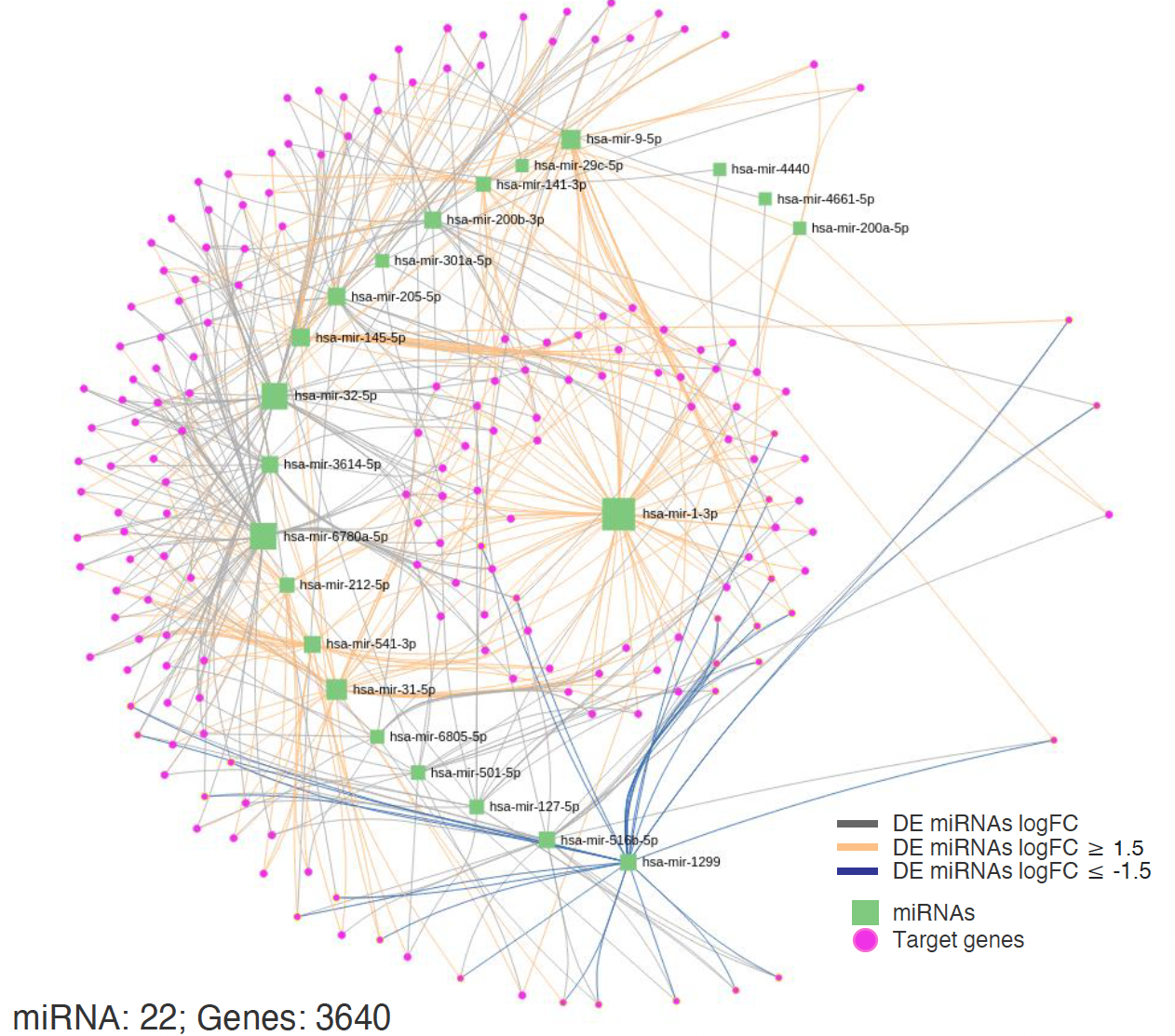


**Suppl. Figure 1.**  Differential expressed miRNA predicted target genes. miRNAs’ network of targets predicted by miRNet. Interactions between target genes and miRNAs are shown in orange if the latter were upregulated and in blue if they were downregulated.

**
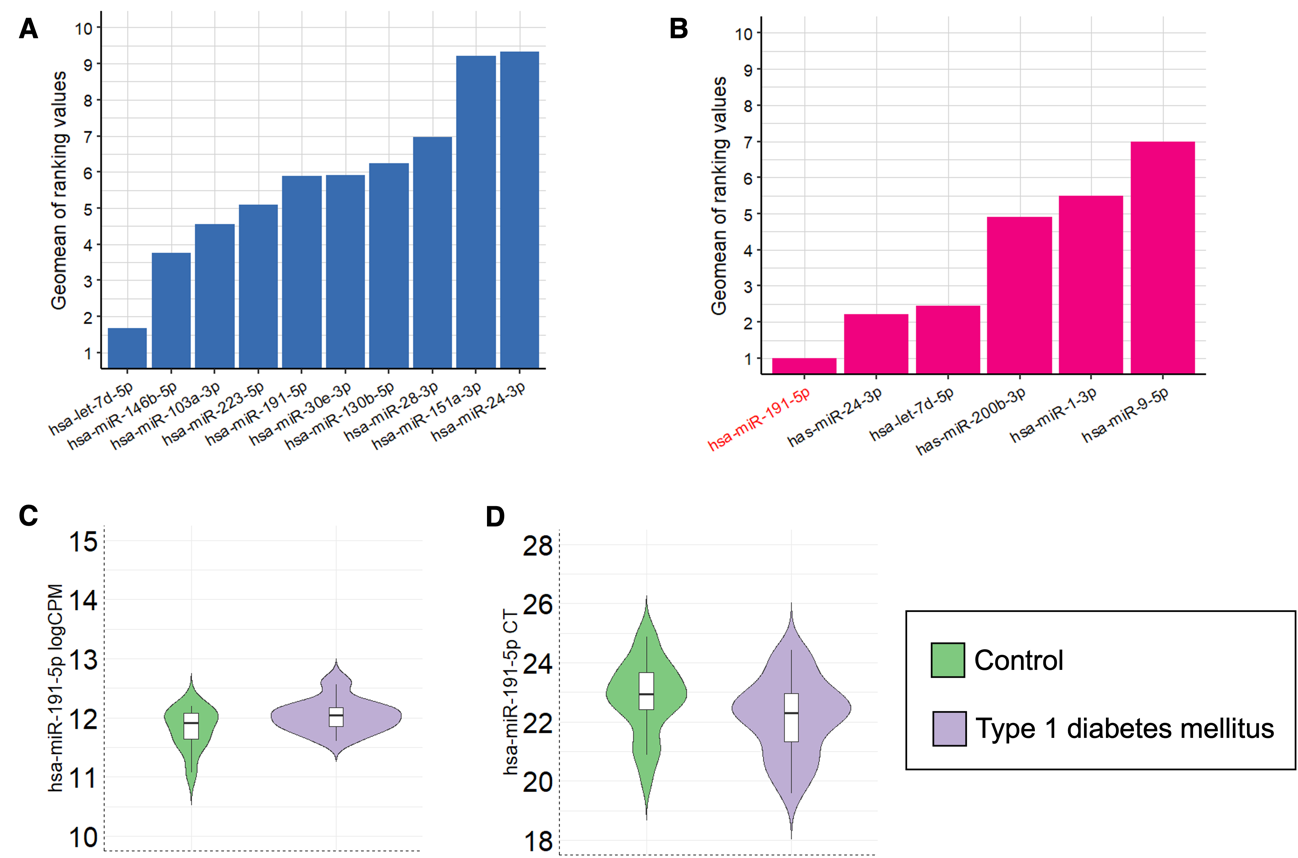
**

**Suppl. Figure 2.** Normalizer miRNA selection by using RefFinder tool. Selected miRNAs is highlighted in red (A) NGS ranking (B) RT-PCR ranking (C-D) Boxplot of selected miRNA normalizer. (C) Next generation sequencing miR-191-3p CPM (D) RT-PCR miR-191-3p expression.
